# Aboveground Biomass and Importance Value of Constructive Species in Desert Steppe Are Co‐Regulated by Grazing Intensity and Climate

**DOI:** 10.1002/ece3.72243

**Published:** 2025-10-03

**Authors:** Aimin Zhu, Qian Wu, Guodong Han, Rui Wang, Bingying Wang, Yan Yang, Ruixia Wang, Lanhua Wu

**Affiliations:** ^1^ College of Grassland Science, Key Laboratory of Grassland Resources, Ministry of Education P.R. of China Inner Mongolia Agricultural University Hohhot China; ^2^ Innovative Team for Grass Germplasm and Sustainable Utilization of Grassland Resources Inner Mongolia Agricultural University Hohhot China; ^3^ Key Laboratory of Forage Cultivation, Processing and Higher Efficient Utilization of the Ministry of Agriculture and Rural Affairs of China Inner Mongolia Agricultural University Hohhot China; ^4^ Inner Mongolia Key Laboratory of Grassland Management and Utilization Inner Mongolia Agricultural University Hohhot China; ^5^ Agricultural, Animal Husbandry and Water Affairs Bureau of Wuda District Wuhai China; ^6^ Bahrain Right Banner Forest and Grassland Protection and Development Center Chifeng China; ^7^ Urad Middle Banner Green Industry Development Center Urad Middle Banner China; ^8^ Urad Middle Banner Bureau of Agriculture, Animal Husbandry, and Science and Technology Urad Middle Banner China

**Keywords:** biomass, constructive species, desert steppe, grazing, importance value, rainfall, temperature

## Abstract

Constructive species are the creators and constructors of plant communities, exerting significant control over community structure and environmental formation. This study investigates the effects of grazing intensity on the biomass and importance value of the constructive species in a desert steppe and its contribution to community biomass. This study conducted a 5‐year monitoring on a long‐term grazing experimental platform in desert grasslands. The experiment set four grazing intensities, and from May to September each year, the aboveground biomass, plant height, density, and community biomass of the community were measured. Results showed that different grazing intensities altered the importance value and aboveground biomass of *Stipa breviflora* , which were regulated by rainfall and temperature during the growing season. In years with higher rainfall, the importance value was relatively low, whereas in years with higher average growing season temperatures, the importance value was higher. In dry years with less rainfall and higher temperatures, the importance value of *S. breviflora* under moderate and heavy grazing approached 1, significantly greater than that under control and light grazing. The effects of grazing intensity on biomass varied across years. In the wet year of 2016, aboveground standing crop increased with grazing intensity, while in dry years (2017) and average rainfall years (2018 and 2019), the trend reversed. Furthermore, compared to the control, light, moderate, and heavy grazing increased the contribution rate of *S. breviflora* aboveground biomass to community biomass. The study concludes that constructive species in desert steppes exhibit greater adaptability and growth advantages under the dual pressures of climate variability and grazing, which intensifies competition with other plants and makes the desert steppe more vulnerable. This phenomenon intensifies with increasing grazing intensity. Therefore, the study suggests that light grazing management in desert steppes is beneficial for adapting to future climate variability and challenges.

## Graphical abstract

This study, through five years of monitoring, investigated the effects of grazing intensity on the biomass and importance value of the constructive species *Stipa breviflora* in desert steppes, as well as its contribution to community biomass. The results showed that grazing intensity altered the importance value and aboveground biomass of *S. breviflora* , which were regulated by rainfall and temperature. As grazing intensity increased, the competitiveness and contribution rate of *S. breviflora* significantly improved, but this also intensified the vulnerability of the desert steppe. The study suggests that light grazing management is beneficial for adapting to future climate variability and challenges.

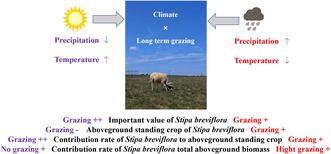

## 1 Introduction

Grazing is one of the primary factors affecting grassland plant productivity and population structure. The impact of grazing on the growth and productivity of grassland plants is multifaceted, among which grazing density (Xu et al. 2007), grazing livestock diet selection, and plant recovery ability (Pastor and Naiman 1992) are considered the main reasons for the impact of grazing on grassland net productivity. Differences in livestock feeding quantity and preferences alter plant community composition, litter quantity and quality, and plant nutrient content, thereby disrupting the normal cycling of materials and energy within the food chain, and this creates a balance between livestock feeding selectivity and plant self‐recovery capacity. However, when this balance is disrupted, it can negatively impact grassland plant communities (Augustine and Mcnaughton 1998). Studies have shown that heavy grazing reduces plant diversity and aboveground or belowground productivity (Milchunas and Lauenroth 1993; Ritclue et al. 1998; Han et al. 2007; Savi et al. 2013), primarily because, under heavy grazing, perennial grasses with high grazing tolerance are gradually replaced by annual plants with low grazing tolerance. In severe cases, this may lead to grassland desertification (Breman et al. 1980; Van de Koppel et al. 1997).

In recent years, the response mechanisms of constructive species in grassland communities to grazing have emerged as a focal topic in grassland ecology research. Constructive species play a critical role in maintaining structural stability, resource‐use efficiency, and ecological functions of grassland ecosystems (Zhu et al. 2021). Empirical studies have demonstrated that moderate grazing facilitates increased activities of nitrogen assimilation enzymes in dominant species, thereby enhancing their nutrient absorption and utilization capacities, which, in turn, promotes growth and sustains community stability (Muhammad et al. 2023; Wang et al. 2024). Conversely, intensive grazing leads to a significant decline in the biomass of dominant species, compromising community stability and resistance to disturbance (Huo et al. 2025). Moreover, the responses of constructive species to grazing differ among grassland types. In meadow steppes, moderate grazing helps maintain the dominance of key species and promotes community diversity. In contrast, in desert steppes, heavy grazing results in reductions in both biomass and importance value of constructive species, leading to a simplification of community structure (Lv et al. 2024).

Plant population characteristics generally reflect changes in plant growth patterns, reproductive strategies, and more. While grazing affects aboveground productivity and diversity, it also alters the spatial distribution (Adler et al. 2001), morphological characteristics (Wang et al. 2000a, 2000b; Gallacher and Hill 2006), and nutrient allocation (Louahlia et al. 2000) of plant populations. For instance, previous research has found that overgrazing causes grassland plants to exhibit “miniaturization” traits, with plant height reduced by up to 80% compared to ungrazed sites (Dong et al. 2010). This indicates that plants respond to grazing stress by adopting adaptive strategies such as reduced height, smaller organs, and increased density, as well as altering their nutrient absorption and utilization efficiency. In research, the importance value is commonly employed as a comprehensive indicator to assess species' growth status and their relative significance within plant communities.

The impact of grazing on plant productivity and population characteristics is a complex process. While many argue that herbivores have a negative relationship with plant growth (Frank et al. 1998), other theories suggest that grazing may elicit neutral or positive responses in primary productivity at both individual and ecosystem levels, as proposed in the grazing optimization hypothesis (Hilbert et al. 1981; Knapp et al. 2012). For example, studies conducted on German calcareous grasslands indicate that low‐intensity livestock grazing can markedly enhance aboveground biomass, thereby supporting both agricultural productivity and biodiversity conservation (Kleinebecker et al. 2011). Research in typical grasslands of Inner Mongolia indicates that light or moderate grazing enhances aboveground net primary productivity (Schonbach et al. 2011). Soil nutrient content is a critical factor influencing grassland plant productivity and plays a decisive role in healthy plant growth. However, grazing‐induced compensatory plant growth is influenced by multiple factors, including climate and plant characteristics, as well as grazing livestock types, grazing duration, and management practices (Hicks and Reader 1995). Therefore, longer‐term experiments, observations, and analyses are necessary to reveal the mechanisms behind plant adaptation strategies to grazing.

As a fragile ecosystem type, the desert steppe is highly sensitive to grazing. *Stipa breviflora* is a constructive species in the desert steppe ecosystem of China's arid regions. This study monitored the dominant species of a desert steppe subjected to nearly 20 years of grazing history over a five‐year period, aiming to answer two scientific questions: (1) How do grazing intensity and climate factors influence the importance value and biomass of constructive species, and their contribution to community biomass? (2) Does the importance value, biomass, and contribution of constructive species in grazed desert steppe grasslands to community biomass change with variations in annual precipitation and temperature? For these two scientific questions, we propose the following hypotheses: (1) Grazing will reduce the importance value, biomass, and contribution to community biomass of constructive species in desert steppes, and uncertain climatic factors may exacerbate this effect; (2) The importance value, and standing biomass of constructive species in grazed desert steppes may show an increasing trend with higher precipitation or lower temperature. This has significant guiding implications for the sustainable management of grasslands.

## 2 Materials and Methods

### 2.1 Overview of the Experimental Site and Design

The study site is located at the Siziwang Base of the Comprehensive Experimental Demonstration Center, Inner Mongolia Academy of Agricultural and Animal Husbandry Sciences (111°53′41.7″ N, 41°46′43.6″ E, altitude 1456 m). The area belongs to the typical temperate continental climate zone and is representative of the *S. breviflora* desert steppe region. This grazing experiment began in 2004. Based on the theoretical livestock carrying capacity for *S. breviflora* steppe proposed by Wei et al. (2000), grazing rates were controlled in the field over the long term. A randomized block design was employed. The grazing area was divided into three blocks (Block I, Block II, Block III), each containing four treatment plots: no grazing (control, CK), light grazing (LG), moderate grazing (MG), and heavy grazing (HG). Each treatment was replicated three times, and the area of each plot was 4.4 ha. The stocking rates were set as follows: 0 (CK), 0.91 (LG), 1.82 (MG), and 2.71 (HG) sheep units per hectare per half a year. Adult two‐year‐old wether sheep from the Siziwang Banner area were used. The grazing period lasted from June to November each year. Management measures across plots were consistent during grazing, with sheep allowed to graze freely in the plots from 7 a.m. to 6 p.m. daily. Water was provided twice daily, and salt was supplemented regularly using salt blocks. It is worth noting that due to the impact of the COVID‐19 pandemic in 2020, grazing was delayed by two months.

### 2.2 Meteorological Data Monitoring and Collection

Meteorological data for this study were primarily provided by a small weather station (CR1000) established in 2012. The station recorded data automatically every minute, with backups made monthly. Data used in this study were collected between 2016 and 2020, specifically focusing on temperature and rainfall.

**FIGURE 1:**
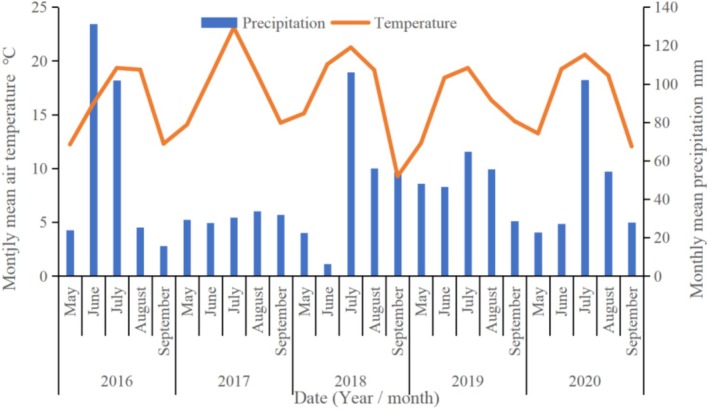
Annual dynamic changes of precipitation and temperature in the experimental sample plot (2016–2020).

During the experimental period (May 1 to September 30) from 2016 to 2020 (Figure 1), the average rainfall in the study area was 234.9 mm. In 2016, rainfall was 63.1 mm above average, classifying it as a wet year. In contrast, 2017 experienced drought conditions, with rainfall 81.9 mm below average. The years 2018, 2019, and 2020 were classified as average rainfall years. The average growing season temperature was 16.7°C, with 2016 having a lower growing season temperature due to higher rainfall, and 2017 experiencing higher temperatures due to drought conditions (Table 1).

**TABLE 1:** Annual rainfall and growing season rainfall of experimental plots from 2016 to 2020.

Year	2016	2017	2018	2019	2020
Yearly total rainfall/mm	337	185.2	273.5	284.2	244.3
Rainfall in growing season (May–September)/mm	298	153	245.4	243.7	234.4
Average temperature in growth season (May–September) /°C	15.8	17.7	16.9	16.2	16.8

### 2.3 Measurement and Analysis of *S. breviflora* Population Characteristics

The determination of aboveground characteristic indicators of grassland plant communities adopts the sampling method. Randomly select 10 0.5 m × 0.5 m plots in each plot, and measure the density and height of each plant species in the plots from May to September each year from 2016 to 2019, and record them. Each grazing treatment is repeated 30 times. After completing the investigation of plant density and height, use an iron rake to pick up the litter and collect it. Then, divide the vegetation into different types and cut them evenly on the ground. Take the aboveground part and dry it in a 65°C oven for 48 h. Weigh the dry weight to obtain the aboveground biomass of different species. The aboveground biomass of the community is the sum of the aboveground biomass of different species. Due to the impact of the COVID‐19 pandemic in 2020, we were unable to collect complete monthly data (May–September) on plant growth conditions in the grazing plots. Therefore, this study only utilized the available August data collected during the peak growing season.

### 2.4 Calculation of Key Indicators

The following equations were used to calculate various indicators for *S. breviflora* and the plant community:

Importance value of *S. breviflora*

$$\begin{array}{c} \text{Importance Value}\\ \;=\frac{\text{Relative Standing Biomass}+\text{Relative Height}+\text{Relative Density}}{3} \end{array}$$

Community aboveground biomass

Community Biomass=Standing Biomass+Litter Biomass

Contribution rate of *S. breviflora* standing biomass to community standing biomass

$$\text{Contribution Rate}=\frac{S.\;\text{breviflora}\;\text{Standing Biomass}}{C\text{ommunity Standing Biomass}}$$

Contribution rate of *S. breviflora* standing biomass to community biomass

$$\text{Contribution Rate}=\frac{S.\;\text{breviflora}\;\text{Standing Biomass}}{\text{Community Biomass}}$$

### 2.5 Data Analysis

Data were analyzed using SPSS 25.0 and R statistical software. One‐way analysis of variance (ANOVA) and Duncan's test were used to evaluate differences among grazing intensity, with significance set at *p* < 0.05. The fitting of rainfall, temperature, important values, and biomass was performed using Sigmaplot 14.0 software.

## 3 Results

### 3.1 Effects of Stocking Rate on Aboveground Standing Biomass of *S. breviflora*

Multivariate ANOVA (Table 2) showed that stocking rate, year, and month had significant effects on the aboveground standing biomass of *S. breviflora* (*p* < 0.05). However, interactions between stocking rate and year or month did not significantly influence standing biomass (*p* > 0.05).

**TABLE 2:** Multivariate analysis of variance of effects of stocking rate, year, month, and their interaction on aboveground biomass of *S. breviflora* .

Treatments	Df	*F* value	Significance
Stocking rate	3	3.300	*p* < 0.05
Year	3	4.120	*p* < 0.05
Month	4	2.779	*p* < 0.05
Stocking rate × Year	9	0.285	*p* > 0.05
Stocking rate × Month	12	0.273	*p* > 0.05

**FIGURE 2:**
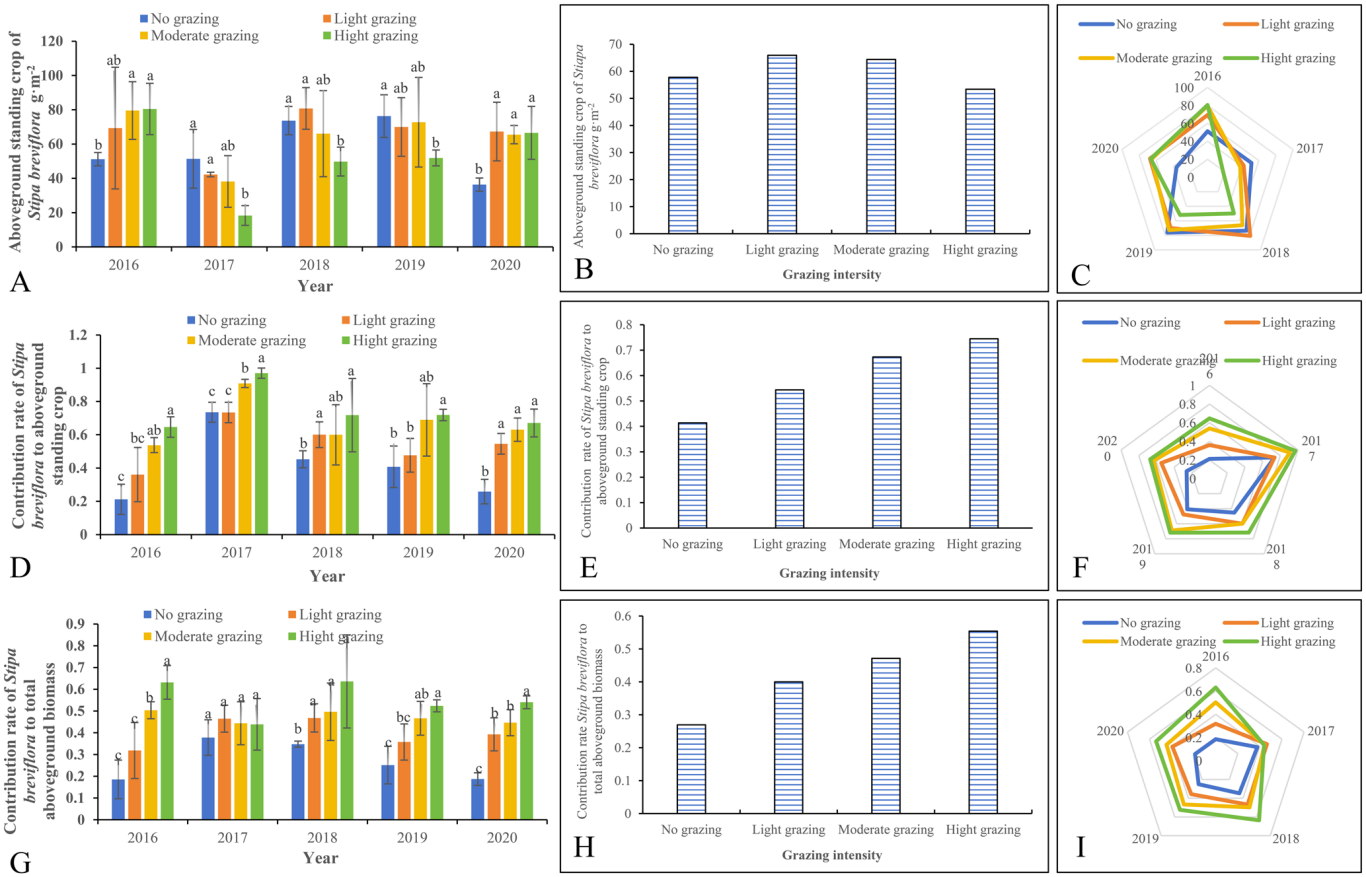
Changes in the standing crop of *S. breviflora* grassland and its contribution to the community's aboveground standing crop and biomass. Different lowercase letters indicate significant differences at the 0.05 level between indicators under different stocking rates. (A) The impact of stocking rates on the aboveground biomass of *S. breviflora* in different years (*n* = 60, One‐way ANOVA); (B) The overall trend of the impact of stocking rates on the aboveground biomass of *S. breviflora* ; (C) Radar chart analysis of the impact of stocking rates on the aboveground biomass of *S. breviflora* in different years; (D) Changes in the contribution rate of *S. breviflora* aboveground biomass to the total aboveground standing crop in different years (*n* = 60, One‐way ANOVA); (E) The trend of changes in the contribution rate of *S. breviflora* aboveground biomass to the total aboveground standing crop; (F) Radar chart analysis of the contribution rate of *S. breviflora* aboveground biomass to the total aboveground standing crop in different years; (G) Changes in the contribution rate of *S. breviflora* aboveground biomass to the total aboveground biomass in different years (*n* = 60, One‐way ANOVA); (H) The trend of changes in the contribution rate of *S. breviflora* aboveground biomass to the total aboveground biomass; I: Radar chart analysis of the contribution rate of *S. breviflora* aboveground biomass to the total aboveground biomass in different years. (Total sample size: 600)

The impact of annual precipitation on the aboveground standing biomass of *S. breviflora* varied across different stocking rate treatments (Table 1, Figure 2). In 2016, the desert steppe received an annual precipitation of 337 mm, which was significantly higher than the average annual rainfall, classifying it as a wet year. In this year, the aboveground standing biomass of *S. breviflora* exhibited a gradual increase with higher stocking rates, with the biomass under moderate and HG being significantly greater than that of the control (*p* < 0.05); In 2017, the annual precipitation was only 185.2 mm, much lower than the average, making it a dry year. In this case, the aboveground standing biomass of *S. breviflora* decreased as stocking rate increased, with the control and LG treatments showing significantly higher biomass than the HG treatment (*p* < 0.05); In 2018 and 2019, annual precipitation was close to the average. Under HG, the aboveground standing biomass of *S. breviflora* was significantly lower than that of the control (*p* < 0.05), while no significant differences were observed between light and MG treatments compared to the control (*p* > 0.05). In 2020, the aboveground standing biomass of the constructive species *S. breviflora* in grazed plots was significantly greater than that in ungrazed plots (Figure 2A). Overall, the trend of *S. breviflora* biomass across stocking rates followed a pattern of initial increase and subsequent decrease (Figure 2B). Notably, in the drought year of 2017, the productivity of *S. breviflora* dropped significantly (Figure 2C).

The contribution rate of *S. breviflora* standing biomass to the total aboveground biomass of the community consistently increased with higher stocking rates (Figure 2D,E). In 2016, the contribution rate of *S. breviflora* ranged from 21.2% to 64.6%, with moderate and HG treatments showing significantly higher contributions than the control, and HG being significantly higher than LG (*p* < 0.05); in 2017, the contribution rate ranged from 73.5% to 97.0%, with both moderate and HG treatments significantly higher than the control and LG (*p* < 0.05); in 2018 and 2019, the contribution rate ranged from 40.0% to 72.0%, with HG treatments consistently showing significantly higher contributions than the control (*p* < 0.05). In 2020, the contribution rate ranged from 25.9% to 67.0%, with all grazing treatments being significantly greater than the ungrazed control (*p* < 0.05). The highest contribution rate of *S. breviflora* standing biomass to community biomass occurred in the drought year of 2017 (Figure 2F).

As shown in Figure 2G, in 2016, 2018, and 2019, the contribution rate of *S. breviflora* standing biomass to total community aboveground biomass increased progressively with grazing intensity. In 2016, the contribution rate ranged from 18.5% to 63.1%, with HG being significantly higher than the control, LG, and MG treatments (*p* < 0.05). In 2018, the contribution rate ranged from 34.7% to 63.6%, with light, moderate, and HG treatments all being significantly higher than the control (*p* < 0.05); in 2019, the contribution rate ranged from 25.1% to 52.4%, with moderate and HG treatments significantly higher than the control (*p* < 0.05), and HG significantly higher than LG (*p* < 0.05). In 2020, the contribution rate ranged from 18.7% to 54.0%, with all grazed treatments being significantly greater than the ungrazed control, and HG showed significantly higher than both light and MG treatments (*p* < 0.05). However, in 2017, the contribution rate under different stocking rate treatments did not show significant differences, ranging from 37.8% to 43.9%. Overall, the contribution rate displayed an increasing trend with higher grazing intensity (Figure 2H). Unlike the contribution rate of *S. breviflora* standing biomass to total community biomass, in the dry year of 2017, the contribution rate of *S. breviflora* standing biomass to total community aboveground biomass was the lowest under HG. In contrast, under no grazing, *S. breviflora* showed a relatively higher contribution to community biomass (Figure 2I).

### 3.2 Seasonal Dynamics of Aboveground Standing Biomass of *Stipa breviflora*

**FIGURE 3:**
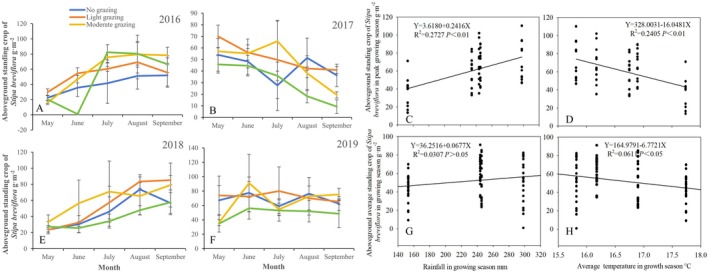
Seasonal dynamic change of the effect of stocking rate on aboveground standing stock of *S. breviflora* (2016–2019). (A, B, E, F) Represent the impact of stocking rates on the aboveground standing crop of *S. breviflora* in different seasons across various years; (C, D) represent the fitting curves between the aboveground standing crop of *S. breviflora* during the peak growth period and the rainfall during the growth season, as well as the average temperature during the growth season; G and H represent the fitting curves between the aboveground standing crop of *S. breviflora* during the growth season and the rainfall during the growth season, as well as the average temperature during the growth season.

As shown in Figure 3, under different grazing intensities, the aboveground biomass of *S. breviflora* in 2016 and 2018 was higher in July, August, and September, and lower in May and June (Figure 3A,E). However, in 2017, except for the no‐grazing control treatment, the aboveground biomass of *S. breviflora* was relatively high in May and June and lower in August and September (Figure 3B). In 2019, the seasonal variation in standing biomass was minimal across different months (Figure 3F). Differences among grazing treatments varied by month. For example, in July, August, and September of 2016, the aboveground standing biomass in moderate and HG areas was greater than that in control and LG areas. In contrast, in 2017, 2018, and 2019, the standing biomass of *S. breviflora* was relatively lower under HG.

The variation in the aboveground standing biomass of *S. breviflora* across years and months was significantly related to growing season rainfall and temperature. As shown in Figure 3C,D,G,H, the standing biomass during the growing season (May–August) and peak growing season (August) increased significantly with rising rainfall. The relationships followed the linear equations: *Y* = 3.6180 + 0.2416*X* (*R* ^2^ = 0.2727, *p* < 0.01), *Y* = 36.2516 + 0.0677*X* (*R* ^2^ = 0.0307, *p* > 0.05). This indicates a significant positive correlation between growing season rainfall and peak standing biomass of *S. breviflora* . However, the relationship between growing season rainfall and average standing biomass did not reach statistical significance. With rising temperatures, both peak growing season and growing season standing biomass of *S. breviflora* decreased, following the linear equations: *Y* = 328.0031–16.0481*X* (*R* ^2^ = 0.2405, *p* < 0.01), *Y* = 164.9791–6.7721*X* (*R* ^2^ = 0.0611, *p* < 0.05). This demonstrates significant negative correlations between temperature and *S. breviflora* standing biomass during both periods.

### 3.3 Effects of Grazing on the Importance Value of *S. breviflora*

The importance value is a key indicator reflecting the relative importance of plant species in a community. Results from multivariate ANOVA (Table 3) show that stocking rate, year, and month all had highly significant effects on the importance value of *S. breviflora* (*p* < 0.01). However, the interactions between stocking rate and year or month did not significantly affect the importance value of *S. breviflora* (*p* > 0.05).

**TABLE 3:** Multivariate analysis of variance of the effects of stocking rate, year, month, and their interaction on the important value of *S. breviflora* .

Treatments	Df	*F* value	Significance
Stocking rate	3	22.295	*p* < 0.01
Year	3	13.465	*p* < 0.01
Month	4	4.624	*p* < 0.01
Stocking rate × Year	9	0.285	*p* > 0.05
Stocking rate × Month	12	0.273	*p* > 0.05

**FIGURE 4:**
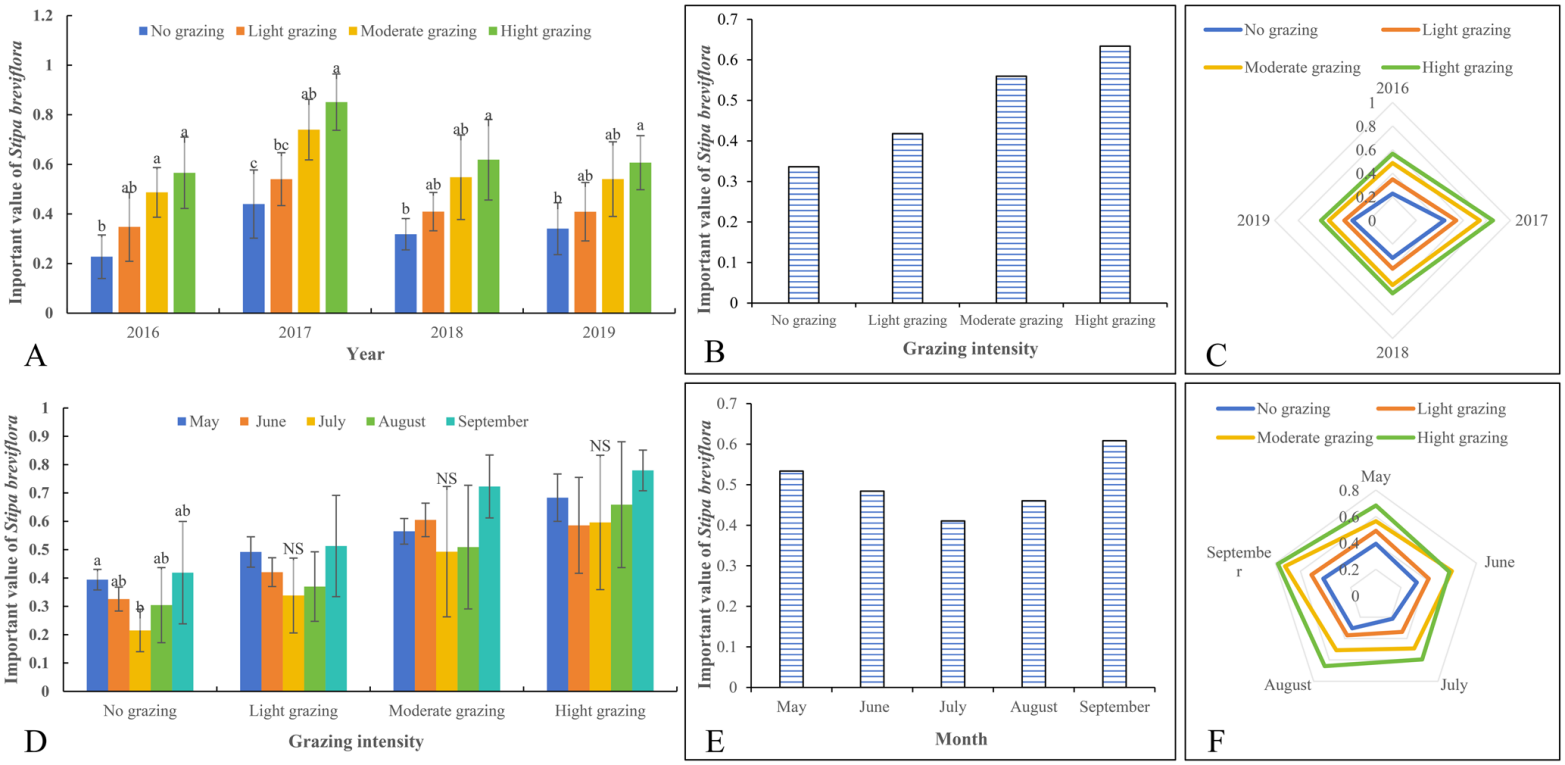
Effect of stocking rate on important value of *S. breviflora* (2016–2019). Different lowercase letters represent significant differences between treatments or months at the level of 0.05. (A) The impact of stocking rates on the importance value of *S. breviflora* in different years (*n* = 48, One‐way ANOVA); (B) The overall trend of the impact of stocking rates on the importance value of *S. breviflora* ; (C) Radar chart analysis of the impact of stocking rates on the importance value of *S. breviflora* in different years; (D) Changes in the importance value of *S. breviflora* between months under different stocking rates (*n* = 60, One‐way ANOVA); (E) The overall trend of changes in the importance value of *S. breviflora* between months; (F) Radar chart analysis of the impact of stocking rates on the importance value of *S. breviflora* between months (Total sample size: 480).

From 2016 to 2019, the importance value of *S. breviflora* gradually increased with grazing intensity (Figure 4A,B). In 2016 and 2017, the importance value of *S. breviflora* ranged from 0.228 to 0.566 and from 0.440 to 0.851, respectively, with HG significantly greater than the control (*p* < 0.05). In 2018 and 2019, the importance value ranged from 0.318 to 0.619 and from 0.341 to 0.607, respectively. HG still exhibited significantly higher values than the control (*p* < 0.05), while differences between light, moderate, and control treatments were not significant (*p* > 0.05). A radar chart indicates that the importance value of *S. breviflora* was higher in 2017 compared to other years (Figure 4C), likely due to lower rainfall (< 200 mm) that year.

Monthly comparisons from 2016 to 2019 revealed a V‐shaped pattern in the importance value of *S. breviflora* from May to September (Figure 4D–F). The importance value was lower in July and August but higher in May and September.

### 3.4 Seasonal Dynamic Changes of Important Values of *S. breviflora*

**FIGURE 5:**
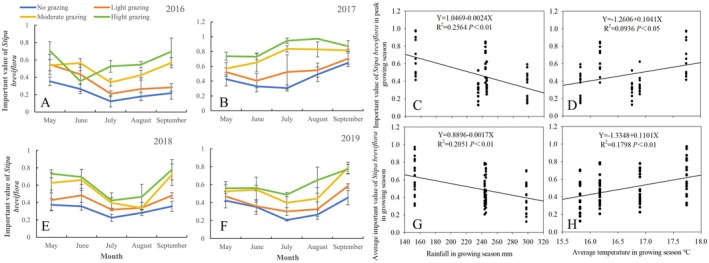
Seasonal dynamic changes in the importance value of *S. breviflora* under different grazing intensities and the fitted curves with growing season precipitation and temperature (2016–2019). (A, B, E, F) Represent the impact of stocking rates on the importance value of *S. breviflora* in different seasons across various years; (C, D) represent the fitting curves between the importance value of *S. breviflora* during the peak growth period and the rainfall during the growth season, as well as the average temperature during the growth season; (G, H) represent the fitting curves between the importance value of *S. breviflora* during the growth season and the rainfall during the growth season, as well as the average temperature during the growth season.

From May to September (2016–2019), the importance value of *S. breviflora* followed the trend: HG > MG > LG > control, except for June 2016 (Figure 5A,B,E,F). In 2016 and 2018–2019, the importance value of *S. breviflora* reached its lowest point in July for all grazing treatments, with a V‐shaped seasonal trend (Figure 5A,E,F). In 2017, the importance value of *S. breviflora* under moderate and HG peaked in July and August, respectively, significantly exceeding that of LG and control (*p* < 0.05). For LG and control, the lowest importance values were observed in June and July, respectively (Figure 5B).

The differences in the importance value across years and months were influenced by growing season rainfall and temperature. As shown in Figure 5C,D,G,H, the importance value of *S. breviflora* during the growing season (May–August) and peak growing season (August) decreased significantly with increasing rainfall. The relationships followed the linear equations: *Y* = 1.0469–0.0024*X* (*R* ^2^ = 0.2564, *p* < 0.01), *Y* = 0.8896–0.0017*X* (*R* ^2^ = 0.2051, *p* < 0.01). This indicates a highly significant negative correlation between the importance value and average importance value of *S. breviflora* during the growing season and the rainfall during the growing season; and the importance value increased significantly with rising temperatures during both periods, following the linear equations: *Y* = −1.2606 + 0.1041*X* (*R* ^2^ = 0.0939, *p* < 0.05), *Y* = −1.3348 + 0.1101*X* (*R* ^2^ = 0.1789, *p* < 0.01). This indicates a significant positive correlation between importance values of *S. breviflora* and the temperature during the growing season.

### 3.5 Effects of Climate and Grazing Disturbance on the Importance Value and Biomass of Dominant Species

**FIGURE 6:**
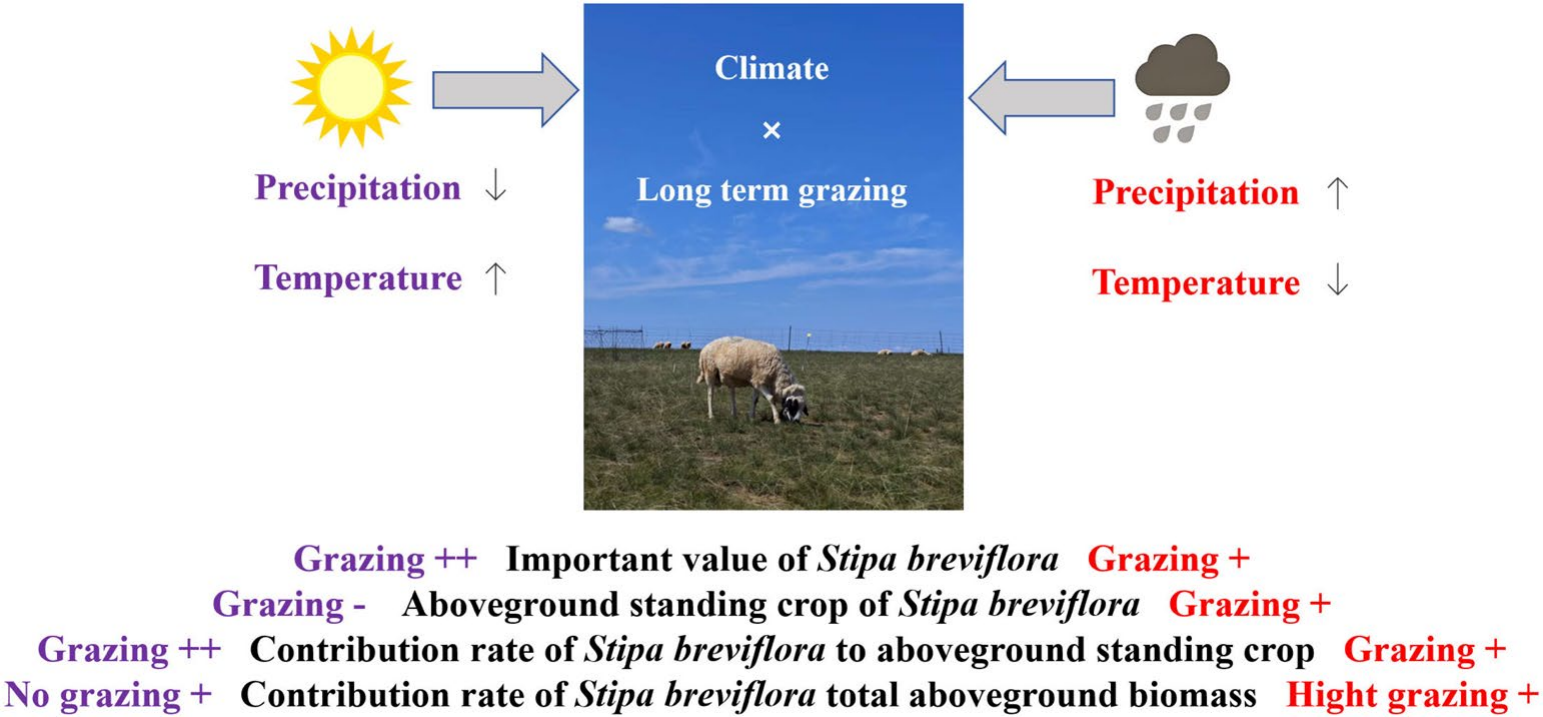
The aboveground biomass, importance value, and contribution to community biomass of constructive species in desert grasslands are jointly regulated by grazing intensity and climate. The purple color in the figure represents the impact of grazing on the importance value, biomass, and contribution to the community of constructive species in drought years (with reduced rainfall and increased temperature); The red color represents the impact of grazing on the importance value, biomass, and contribution to the community of established species in rainy years (with increased rainfall and decreased temperature). In addition, “+” represents that climate and grazing have a positive impact on the indicator, “−” represents that climate and grazing have a negative impact on the indicator, and “++” represents that climate and grazing have a significant positive impact on the indicator.

Based on our research results, as shown in Figure 6, we summarize the performance of desert grassland constructive species under different grazing intensities in dry and rainy years. Long‐term grazing in the desert steppe increased the importance value and contribution rate of constructive species to community biomass during both drought and wet years, and the increase was much more pronounced in drought years compared to other years. During drought and normal rainfall years, grazing reduced the aboveground standing biomass of constructive species, whereas in wet years, grazing increased aboveground biomass.

Under no‐grazing conditions, the contribution rate of *S. breviflora* to community biomass was significantly higher in drought years compared to other years. In wet years, HG increased the contribution rate of *S. breviflora* standing biomass to community biomass.

## 4 Discussion

Scientifically evaluating the health status of grassland communities is a critical part of developing sustainable grassland management strategies (Liu and Shao 2024), especially in desert steppe ecosystems that are sensitive to grazing and have harsh climatic conditions. How does long‐term grazing affect the population characteristics of constructive species in grasslands? The results of this study indicate that grazing increases the importance value of the constructive species *S. breviflora* , and the higher the grazing intensity, the greater the importance value. In the drought year of 2017, the importance value of *S. breviflora* under various grazing intensities was higher than in other years with the same grazing treatments. The aboveground biomass of *S. breviflora* generally showed a trend of first increasing and then decreasing with rising grazing intensity. However, this pattern was modulated by precipitation and temperature. For instance, in the relatively dry year of 2017, the importance value of *S. breviflora* declined with increasing grazing intensity. Moreover, the contribution of *S. breviflora* to aboveground biomass under different grazing intensities was greater in 2017 than in other years. These findings suggest that Hypothesis 1 is not supported. Additionally, the aboveground biomass of the constructive species tended to increase with growing season precipitation and decrease with growing season temperature, while the trend in importance value was opposite. This result indicates that Hypothesis 2 is also not supported.

This study demonstrates that the aboveground biomass, importance value, and contribution of constructive species in desert steppe ecosystems are co‐regulated by grazing intensity and climate (precipitation and temperature). Overall, grazing reduced the aboveground standing biomass of *S. breviflora* in dry and average rainfall years but increased its standing biomass in wet years. This finding is inconsistent with the results reported by Cai et al. (2024), whose study demonstrated that in years with sufficient precipitation, MG can enhance the biomass of constructive species and promote community stability. In contrast, during drought years, grazing may exacerbate growth stress on constructive species, leading to a decline in their biomass and a degradation of community structure.

The interactions between grazing and environmental factors such as climate and soil are complex. This study found that in dry years, HG significantly reduced the aboveground standing biomass of *S. breviflora* compared to control and LG, while in wet years, HG significantly increased *S. breviflora* biomass. These findings align with those of Sternberg et al. (2000) and Anderson et al. (2007), who reported similar results. The main reason is that long‐term HG, while reducing the size of *S. breviflora* clumps, increases plant density and broadens spatial distribution. During wet years, the aboveground productivity of *S. breviflora* increases rapidly. Furthermore, *S. breviflora* biomass exhibits significant temporal fluctuations because productivity during the early growth period is limited by temperature, whereas productivity during the entire growing season is predominantly influenced by rainfall. Additionally, during July and August, when both temperature and rainfall are high, annual plants grow rapidly, competing with *S. breviflora* for resources. This reduces the importance value and contribution of *S. breviflora* to community biomass. Meanwhile, rainfall after June and cooler temperatures after August positively and negatively impact the accumulation of *S. breviflora* aboveground productivity, respectively. These findings indicate that the variation in aboveground biomass of desert steppe plants is influenced not only by grazing but also by abiotic factors such as temperature and rainfall.

This study also found that grazing increased the importance value of *S. breviflora* across all years, regardless of rainfall levels, with the importance value rising significantly with higher grazing intensity. In this study, the importance value was calculated based on plant height, density, and standing biomass. The selection of these three indicators was justified as follows: the aboveground biomass of *S. breviflora* contributes significantly to the total standing biomass of the community, and standing biomass is an important metric for evaluating the sustainable use of grasslands. Plant height was included because previous studies have shown that higher grazing intensity leads to “miniaturization” of plants, which is associated with reduced aboveground biomass (Wang et al. 2000a, 2000b; Gallacher and Hill 2006). Plant density was included because *S. breviflora* exhibits increased spatial distribution uniformity, niche breadth, and inter‐plant competition under higher grazing intensities (Lv et al. 2017). The dominant role of *S. breviflora* in areas with low density also diminishes, and its density undergoes significant changes. This study showed that across all years—wet, average, and dry—the importance value of *S. breviflora* increased with grazing intensity, approaching 1 under HG during dry years. This phenomenon is primarily attributed to long‐term grazing altering the community structure of desert steppe plants. In areas with HG, annual and biennial plants are only widely distributed during years with sufficient rainfall and are scarce in dry years. This limits the aboveground biomass and density of the plant community. Additionally, long‐term monitoring revealed that the aboveground productivity and density of shrubs and semi‐shrubs in moderately and heavily grazed areas declined at varying levels, further affecting the importance value of *S. breviflora* .

Furthermore, this study indicates that in dry years, due to high temperatures and limited rainfall, the contribution of *S. breviflora* biomass to community biomass was highest under no grazing and lowest under HG. This suggests that the growth of *S. breviflora* is more inhibited by the combined effects of drought and HG compared to no, light, or MG. Conversely, in wet years, abundant rainfall led to vigorous growth of *S. breviflora* , resulting in higher aboveground biomass in grazed areas than in ungrazed areas. This phenomenon may represent an adaptive survival strategy of *S. breviflora* in response to climate change and grazing disturbance. Plant resource acquisition strategies influence their responsiveness to grazing (Streit et al. 2022). Under HG, non‐selective feeding and intense trampling intensify environmental filtering effects, resulting in a simplification of community structure. Consequently, dominant species gain a greater advantage in resource acquisition (Guo et al. 2025). There is limited prior research on quantifying the contribution of constructive species' biomass and importance value to community biomass, which warrants further investigation. This represents an important scientific question for future ecological research.

## 5 Conclusion

The desert steppe is highly sensitive to both climate change and grazing pressure. Although the constructive species *S. breviflora* exhibits strong adaptability and competitive advantages, this suppresses the growth of other species, leading to increased ecosystem fragility, particularly under HG conditions. This study recommends implementing an LG regime to protect this vulnerable ecosystem and promote sustainable grassland utilization under climate warming.

## Author Contributions

**Aimin Zhu:** data curation (equal), funding acquisition (supporting), writing – original draft (lead), writing – review and editing (lead). **Qian Wu:** data curation (equal), formal analysis (equal). **Guodong Han:** conceptualization (lead), data curation (equal), formal analysis (equal), funding acquisition (supporting), investigation (equal), methodology (lead), project administration (equal), resources (lead), supervision (equal), validation (equal), visualization (equal). **Rui Wang:** data curation (equal). **Bingying Wang:** data curation (equal), methodology (equal). **Yan Yang:** data curation (equal), formal analysis (equal). **Ruixia Wang:** data curation (equal), formal analysis (equal). **Lanhua Wu:** data curation (equal), formal analysis (equal).

## Ethics Statement

The authors have nothing to report.

## Consent

The authors have nothing to report.

## Conflicts of Interest

The authors declare no conflicts of interest.

## Supporting information


**Data S1:** ece372243‐sup‐0001‐supinfo.xlsx.

**Data S2:** ece372243‐sup‐0002‐supinfo.xlsx.

**Data S3:** ece372243‐sup‐0003‐supinfo.xlsx.

**Data S4:** ece372243‐sup‐0004‐supinfo.xlsx.

**Data S5:** ece372243‐sup‐0005‐supinfo.xlsx.

**Data S6:** ece372243‐sup‐0006‐supinfo.xlsx.

**Data S7:** ece372243‐sup‐0007‐supinfo.xlsx.

## Data Availability

The data has been uploaded as Supporting Information.
